# RNA Deep Sequencing Analysis Reveals That Nicotine Restores Impaired Gene Expression by Viral Proteins in the Brains of HIV-1 Transgenic Rats

**DOI:** 10.1371/journal.pone.0068517

**Published:** 2013-07-16

**Authors:** Junran Cao, Shaolin Wang, Ju Wang, Wenyan Cui, Tanseli Nesil, Michael Vigorito, Sulie L. Chang, Ming D. Li

**Affiliations:** 1 Department of Psychiatry and Neurobehavioral Sciences, University of Virginia, Charlottesville, Virginia, United States of America; 2 School of Biomedical Engineering, Tianjin Medical University, Tianjin, China; 3 The First Affiliated Hospital, Zhejiang University, Hangzhou, China; 4 Institute of NeuroImmune Pharmacology, Seton Hall University, South Orange, New Jersey, United States of America; 5 Department of Psychology, Seton Hall University, South Orange, New Jersey, United States of America; 6 Department of Biological Sciences, Seton Hall University, South Orange, New Jersey, United States of America; Temple University School of Medicine, United States of America

## Abstract

Persons infected with HIV-1 often develop neurologic disorders despite receiving highly active anti-retroviral therapy. Although the underlying mechanism is largely undetermined, our previous RNA-seq-based study showed that the expression of many genes was altered in the central nervous system (CNS) of HIV-1 transgenic (HIV-1Tg) rats. Because nicotine, a natural agonist of nicotinic acetylcholine receptors, exhibits a neuroprotective effect, we presently tested the hypothesis that nicotine restores the expression of altered genes in the CNS of HIV-1Tg rats. Adult male HIV-1Tg and F344 control strain rats were injected with either nicotine (0.25 mg/kg) or saline subcutaneously twice a day for 17 days. Gene expression in the prefrontal cortex (PFC), dorsal hippocampus (HIP), and dorsal striatum (STR) was evaluated using the RNA deep sequencing technique. We found that about 20% of the altered genes in the HIV-1Tg rat were affected by nicotine in each brain region, with the expression of most restored. Analysis of the restored genes showed distinct pathways corrected by nicotine in different brain regions of HIV-1Tg rats. Specifically, the two most significantly restored pathways were Wnt/β-catenin signaling and ephrin B signaling in the PFC, cAMP-responsive element-binding protein (CREB) signaling and glutathione metabolism pathway in the HIP, and tricarboxylic acid (TCA) cycle and calcium signaling in the STR. Together, our findings indicate that cholinergic modulators such as nicotine have beneficial effects on HIV-1-induced neurologic deficits.

## Introduction

The central nervous system (CNS) is highly vulnerable to infection by the human immunodeficiency virus-1 (HIV-1) [1]. Such an infection induces many neurologic problems, including gliosis, striatal pathology, motor dysfunction, HIV-associated dementia (HAD), and HIV-associated neurocognitive disorder (HAND) [2]–[5]. The neurologic deficits generally are unaffected by highly active anti-retroviral therapy (HAART) although HAART has significantly reduced the morbidity and mortality rate of HIV-positive patients [4]–[6]. In fact, the prevalence of these neurocognitive deficits has increased with the introduction of HAART because the lifespan of HIV-infected patients has been prolonged [5], [6]. Thus, efficient therapies for the HIV-associated neurologic disorders are greatly needed. Although it is still not clear what mechanisms underlie those neurologic disorders, the persistent presence of HIV-1 viral proteins in the host appears to result in brain damage [7]–[9]. Given that HAART can inhibit viral entry and replication without eliminating the viral proteins, the HAART-defying neurologic disorders are very likely to result from these proteins [7], [10]–[13].

The HIV-1 transgenic (HIV-1Tg) rat was developed as a model to investigate the effects of HIV-1 on the CNS and to facilitate therapy development. This rat carries a *gag-pol*-deleted HIV-1 genome under the control of HIV-1 viral long terminal repeats (LTRs), and expresses seven of the nine HIV-1 viral proteins without viral replication [14]. The HIV-1Tg rat develops characteristics similar to those of HIV-1 infected humans, including neurobehavioral and neuropathologic changes [14], deficits in learning and memory [15], [16], and abnormal sensitivity to many psychostimulants [17]–[19]. Thus, this animal model appears to mimic the condition of HIV-1 patients given HAART, who have little viral replication, but persistent HIV-1 brain infection, often associated with slow progressive neurodegeneration.

The cholinergic system plays an important role in cognition [20], [21]. Increased cholinergic transmission in response to agonists of acetylcholine receptors or cholinesterase inhibitors improves cognitive processes in neuropsychiatric disorders, such as Alzheimer’s disease (AD) and schizophrenia [21]–[24]. Nicotine is an agonist of the nicotinic acetylcholine receptors (nAChRs) and the major psychoactive component of cigarettes. Many studies have indicated that nicotine may have neuroprotective effects against cognitive disorders [25]–[27]. For example, smokers have a lower risk of developing AD, Parkinson’s disease (PD), and other neurodegenerative disorders than non-smokers [28], [29], although the effect on AD is still in debate [30]. Nicotine also exhibits neuroprotective effects in several, although not all, animal models of neurodegenerative diseases [31]. In addition to its neuroprotective effects, nicotine functions as an important modulator of immune responses [32]–[35]. Considering these properties, cholinergic modulators such as nicotine represent a potential treatment for neurologic disorders in HIV-1-positive patients.

A few studies have demonstrated an interaction between the cholinergic system and HIV-1 pathogenesis. Gp120, one of the HIV-1 proteins, binds to nAChRs [36]. This protein induces defects in memory, which can be reversed by hippocampal cholinergic stimulation [37]. Further, gp120-induced inflammatory responses can be attenuated by administration of α7 nAChR agonists [38]. Although some studies report that nicotine stimulates HIV-1 production in macrophages [39], [40], HIV-1 proteins and nicotine seem to have opposite effects on their shared pathways, including those mediated by MAPKs and NF-κB. For example, nicotine suppresses gp120-induced microglial activation by inhibiting p44/p43 MAPK activity [41]. HIV-1 proteins decrease cAMP-responsive element binding protein (CREB) phosphorylation, whereas nicotine increases CREB phosphorylation in the prefrontal cortex (PFC) of HIV-1Tg rats [18]. Behaviorally, HIV-1 proteins attenuate nicotine-induced behavioral sensitization [18], while nicotine prevents gp120-induced motor disturbances [42].

Together, these studies suggest that cholinergic modulators have some beneficial effects on the neurologic complications in HIV-1 infected individuals. However, the underlying molecular mechanisms are poorly understood, as only a few genes and pathways have been evaluated with conventional molecular techniques. Thus, the primary objective of this study was to systematically determine whether altered gene expression in HIV-1Tg rats could be restored by nicotine treatment. To achieve our objective, we used the recently developed RNA deep sequencing technique, which can evaluate gene expression in a high-throughput manner, and thus, is capable of providing a systematic view of the effects of nicotine on gene expression in the brains of HIV-1Tg rats.

## Materials and Methods

### Animals

The Animal Care and Use Committee of both the Seton Hall University and University of Virginia approved this study. Adult male rats of HIV-1Tg and F344 background control strain, were purchased from Harlan Inc. (Indianapolis, IN). All rats were double housed in standard plastic rat cages, maintained in a temperature-controlled environment with a 12 h light/dark cycle, and fed a standard rat diet and water *ad libitum*. The animals were monitored daily, and their cage/bedding was changed twice a week. All animals had been used in a previously reported behavioral study [43]. All experimental procedures were conducted during the light cycle in accordance with the Animal Care and Use Committee regulations of each participating institution.

### Nicotine Preparation and Treatment

Nicotine free base (Sigma Aldrich Co., St. Louis, MO) was diluted in 0.9% sterile saline, and the pH adjusted to 7. A fresh stock of nicotine solution was prepared daily. Four groups of rats (n = 12 in each group) were tested: HIV-1Tg Nicotine (HIV_Nic), HIV-1Tg Saline (HIV_Sal), F344 Nicotine (F344_Nic), and F344 Saline (F344_Sal). Either nicotine (0.25 mg/ml) or saline (1 ml/kg of body weight) was administered twice a day (7∶00 AM and 4∶00 PM) by subcutaneous injection with a 23-gauge/1-cc syringe for 17 days. This drug treatment paradigm was based on a previous behavioral study [43].

### Tissue Collection

Using a rat brain matrix, 1 mm slices were taken from each brain, and the slices that contained the prefrontal cortex (PFC), dorsal hippocampus (HIP), and dorsal striatum (STR) were identified according to a rat brain atlas [44]. Tissue from the interested regions was collected bilaterally from each brain using a 3 mm Harris Micro-Punch (GE Healthcare Life Sciences, Piscataway, NJ, USA) and stored at −80°C until use.

### RNA Extraction and Sample Preparation

Total RNA was extracted from each tissue sample using TRIzol (Life Technologies, Grand Island, NY) according to the manufacturer’s protocol. The RNA concentration and quality of each sample were assessed using the Qubit RNA BR Assay Kit (Life Technologies) and the Agilent Bioanalyzer 2100 (Agilent, Santa Clara, CA), respectively.

### RNA Deep Sequencing

RNA deep sequencing was conducted as described previously [45]. Briefly, the sequencing library of each RNA sample was prepared with the TruSeq RNA Sample Preparation Kit according to the manufacturer’s instructions (Illumina, San Diego, CA). The enriched libraries were diluted with elution buffer to a final concentration of 10 nM. Each sample (7 pM) was subjected to 50 cycles of sequencing from both ends in one lane of an Illumina Hiseq2000 Sequencer. Following deep sequencing analysis of 50 bp paired-end reads, Bowtie/Tophat/Cufflinks suites were used to align the reads into transcripts based on the rat reference genome (Rn4) and to measure the relative abundance of each transcript. Expression of each transcript was quantified as the number of reads mapping to a gene divided by the gene length (in kb) and the total number of mapped reads (in millions), and designated as fragments per kilobase of exon per million fragments mapped (FPKM).

### Gene Annotation and Expression Profiling Analysis

The Ensembl Transcript ID was used as the primary identifier for all analyses. When multiple splice variants existed, all of them were selected. In generating the FPKM distributions of intergenic regions, regions with a distance of at least 10 kb from any RefSeq or Ensembl gene were selected. The annotation information corresponding to each Ensembl Transcript ID was retrieved from the Ensembl database via BioMart (http://www.biomart.org/biomart/martview). To convert the Ensembl Transcript ID to Gene ID, ‘Ensembl gene 66’ was selected for the database and ‘Rattus norvegicus genes’ for the dataset.

For each brain region of interest, all the transcripts were pulled from the file generated by Cufflinks. The measurements with RPKM values close to zero (approximately 5% of the total) were discarded. The RPKM values were logarithmically transformed to base 2, and the measurements of each transcript within an experimental group were subjected to outlier detection [46]. Transcripts with fewer than six valid measurements in either comparison group, after the removal of outliers, were discarded. According to the number of transcripts mapped to a gene, the following three cases were considered: 1) Where a single transcript was mapped to a gene, the corresponding intensities were used in all further analyses; 2) Where there were multiple records for a single transcript in the dataset (≥6 in each group of 12 samples), the intensity values were averaged and treated as one record; and 3) Where multiple transcripts mapped to the same gene, they were treated as independent isoforms in the data analysis steps.

A two-way ANOVA (strain × sex) was conducted for each gene in each brain region. For genes showing significant major factor differences or interactions, gene expression was further analyzed by a Tukey-Kramer post-hoc analysis. All analyses were conducted with MATLAB (The Mathworks Inc., Natick, MA). Significance was defined as p<0.05, with a fold change (FC) larger than 20%, as in previous analyses of this type of data [47]–[49].

### Enriched Biochemical Pathways Restored in HIV-1Tg Rats

The genes significantly restored by nicotine in the HIV-1Tg rats were further analyzed using the Ingenuity Pathway Analysis program (IPA; https://analysis.ingenuity.com) in order to identify the biochemical pathways affected. The core component of IPA is the Ingenuity Pathways Knowledge Base (IPKB), which contains the biological function, interaction, and other related information of a curated gene set and more than 330 biochemical pathways. This pathway-based software is designed to identify global canonical pathways, dynamically generated biological networks, and global functions from a given list of genes. Basically, the genes, with their symbol or corresponding GenBank accession number or both, were uploaded into the IPA and compared with the genes included in each canonical pathway using the whole gene set of IPKB as the background. All the pathways with one or more genes overlapping the candidate genes were extracted. In IPA, each of these pathways was assigned a *p* value via Fisher’s exact test, which denoted the probability of overlap between the pathway and the input genes.

### Data Access

The deep sequencing RNA data from this study have been deposited in the NCBI SRA database and are accessible through GEO series accession number GSE47474 (http://www.ncbi.nlm.nih.gov/geo/query/acc.cgi?acc=GSE47474).

## Results

### Nicotine’s Effects on Gene Expression in the Brains of HIV-1Tg Rats

We found 439, 505, and 508 differentially expressed genes in the PFC, HIP, and STR, respectively, between saline-treated HIV-1Tg (HIV_Sal) and saline-treated control strain F344 rats (F344_Sal) (Table 1). Of these genes, 89, 115, and 70 in the PFC, HIP, and STR, respectively, were affected by nicotine in the HIV-1Tg rats (HIV_Nic vs HIV_Sal). The mRNA expression of almost all of these genes in the HIV-1Tg rats was restored at least partially by nicotine in the HIV-1 Tg rats. The only exceptions were immunoglobulin-binding protein 1 (Igbp1) in the HIP (F344_Sal: 1.44±0.06 vs HIV_Sal: 3.55±0.41; *p*<0.05; HIV_Sal: 3.55±0.41 vs HIV_Nic: 11.41±0.24; *p*<0.01) and transcription elongation factor A N-terminal and central domain-containing protein 2 (LOC366431) in the STR (F344_Sal: 4.32±0.05 vs HIV_Sal: 3.77±0.05; *p*<0.01; HIV_Sal: 3.77±0.05 vs HIV_Nic: 3.28±0.03; *p*<0.01), in which nicotine worsened their expression in the HIV-1 Tg rats as compared to F344_Sal controls.

**Table 1:** Number of genes differentially expressed in HIV-1Tg rats and affected by nicotine.

Gene Groups		PFC	HIP	STR
A): Genes differentially expressed in HIV-1 Tg rats (HIV _Sal vs. F344_ Sal)		439	505	508
Genes abnormally expressed and further affected by nicotine in HIV-1Tg rats (HIV_Sal vs. F344_Sal & HIV_Nic vs. HIV_Sal)	B): All	89	115	79
	C): Restored by nicotine treatment	89	114	78
	D): Worsened by nicotine treatment	0	1	1

Number of genes with significant expression differences between saline-treated HIV-1Tg rats (HIV_Sal) and F344 rats (F344 _Sal) (Row A). Some of these genes were further altered by nicotine treatment in HIV-1 Tg rats (Row B), with most restored by nicotine treatment (Row C); only two genes had their expression worsened by nicotine (Row D) in the prefrontal cortex (PFC), dorsal hippocampus (HIP), or dorsal striatum (STR) of HIV-1Tg rats.

### Nicotine-restored Pathways in the Brains of HIV-1Tg Rats

**Figure 1:**
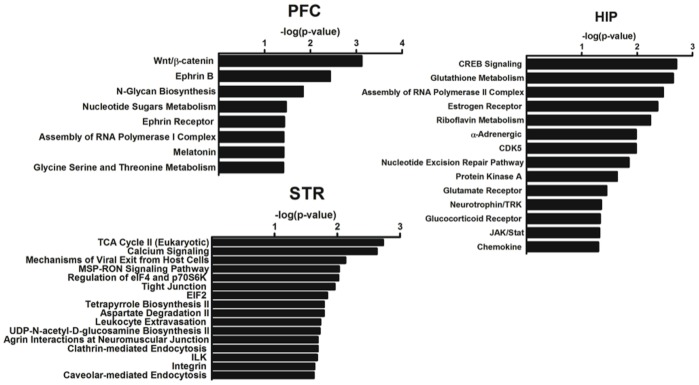
Pathways significantly restored by nicotine in the prefrontal cortex (PFC), dorsal hippocampus (HIP), and dorsal striatum (STR) of HIV-1Tg rats. P-value was calculated using Fisher’s exact test, which denotes the probability of overlap between the pathways and the input genes.

The IPA pathway analysis of the genes restored by nicotine in the HIV-1Tg rats revealed different pathways in distinct brain regions (Figure 1). In the PFC, the pathways significantly restored by nicotine were: Wnt/β-catenin signaling, ephrin B signaling, N-glycan biosynthesis, nucleotide sugars metabolism, ephrin receptor signaling, assembly of RNA polymerase I complex, melatonin signaling, and glycine, serine, and threonine metabolism. In the HIP, the pathways restored by nicotine were CREB signaling, glutathione metabolism, assembly of RNA polymerase II complex, estrogen receptor signaling, riboflavin metabolism, α-adrenergic signaling, CDK5 signaling, nucleotide excision repair pathway, protein kinase A signaling, glutamate receptor signaling, neurotrophin/TRK signaling, glucocorticoid receptor signaling, JAK/stat signaling, and chemokine signaling. In the STR, the nicotine-restored pathways were: TCA cycle II (eukaryotic), calcium signaling, mechanisms of viral exit from host cells, MSP-RON signaling, regulation of eIF4 and p70S6K signaling, tight junction signaling, EIF2 signaling, tetrapyrrole biosynthesis II, aspartate degradation II, leukocyte extravasation signaling, UDP-N-acetyl-D-glucosamine biosynthesis II, agrin interactions at neuromuscular junction, clathrin-mediated endocytosis signaling, and ILK signaling.

### Genes in the Two Most Significantly Restored Pathways in each Brain Region

**Figure 2:**
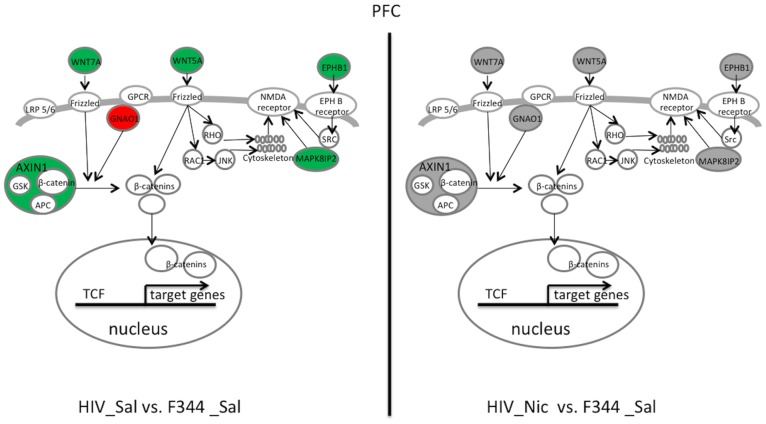
Illustration of top two pathways and related genes restored by nicotine in the prefrontal cortex (PFC) of HIV-1Tg rats. Restored genes and pathways in saline-treated (HIV_Sal, left panel) and nicotine-treated (HIV_Nic, right panel) HIV-1Tg rats are shown. Compared with saline-treated F344 (F344_Sal) control rats, genes shown in red were increased, those shown in green were decreased, and those in gray had similar mRNA expression. APC, adenomatous polyposis coli; AXIN1, Axin-1; EPHB1, Eph receptor B1; Frizzled, frizzled receptor; GNAO1, guanine nucleotide-binding protein G(o) subunit alpha; GPCR, G protein-coupled receptor; GSK, glycogen synthase kinase; JNK, c-Jun N-terminal kinase, LRP5/6, low density lipoprotein receptor-related protein; MAPK8IP2, mitogen-activated protein kinase 8-interacting protein 2; RAC1, ras-related C3 botulinum toxin substrate 1; RHO, rhodopsin; SRC, v-src sarcoma; TCF, transcription factor; WNT5A, wingless-related MMTV integration site 5A; WNT7A, wingless-type MMTV integration site 7A.

In the PFC (Figure 2, Table 2), the genes in the Wnt/beta catenin signaling and ephrin B signaling pathways overlapped to some degree. Compared with F344_Sal rats, HIV_Sal rats showed decreased mRNA expression for wingless-related MMTV integration site 5A (*Wnt5a*), wingless-type MMTV integration site 7A (*Wnt7a*), Axin-1 (*Axin1*), fibroblast growth factor 9 (*Fgf9*), Eph receptor B1 (*Ephb1*), and mitogen-activated protein kinase 8 interacting protein 2 (*Mapk8ip2*). Conversely, the HIV_Sal rats showed higher mRNA expression for guanine nucleotide-binding protein G(o) subunit alpha (*Gnao1*) than did F344_Sal rats. The expression of all of these genes was significantly changed by nicotine in the PFC of HIV-1Tg rats, whereas nicotine significantly decreased only the expression of *Mapk8ip2* in F344 rats. After nicotine treatment, the expression of all of these genes was restored to normal in the HIV-1Tg rats, as there was no significant difference detected between HIV_Nic rats and F344_Sal control rats.

**Table 2:** The expression of genes in the top two pathways restored by nicotine in HIV-1Tg rats.

Brain Region	Gene Symbol	Gene Name	F344_Sal	F344_Nic	HIV_Sal	HIV_Nic
**PFC**	**Wnt/beta catenin and Ephrin B signaling**					
	*Wnt5a*	Wingless-related MMTV integration site 5A	0.65±0.10	0.43±0.27	0.36±0.23*	0.72±0.13†
	*Wnt7a*	Wingless-type MMTV integration site 7A	5.59±0.03	5.66±0.03	4.45±0.14*	5.63±0.05†
	*Axin1*	Axin-1	4.46±0.11	4.59±0.09	2.77±0.24*	4.83±0.14††
	*Gnao1*	Guanine nucleotide-binding protein G(o) subunit alpha	53.95±0.06	64.43±0.08	74.42±0.16*	54.34±0.07†
	*Fgf9*	Fibroblast growth factor 9	3.68±0.14	2.89±0.15	1.41±0.37***	2.83±0.10†
	*Ephb1*	Eph receptor B1	7.75±0.02	7.34±0.02	6.82±0.03***	7.31±0.03†
	*Mapk8ip2*	Mitogen-activated protein kinase 8 interacting protein 2	69.83±0.11	27.24±0.41†	11.16±0.08***	34.78±0.32††
**HIP**	**CREB signaling**					
	*Cabp1*	Calcium-binding protein 1	13.45±0.09	9.12±0.08†††	9.86±0.08**	13.02±0.13†
	*Calm3*	Calmodulin 3	516.89±0.12	509.81±0.04	300.72±0.34*	519.39±0.07†
	*Gnal*	Guanine nucleotide binding protein, alpha stimulating, olfactory type	12.32±0.08	7.95±0.23	5.46±0.32**	14.74±0.08†††
	*Ntrk2*	Neurotrophic tyrosine kinase, receptor, type 2	44.77±0.12	58.24±0.07	26.56±0.27*	47.22±0.15†
	*Polr2c*	Polymerase (RNA) II (DNA directed) polypeptide C	23.40±0.05	23.69±0.04	7.50±0.43**	26.10±0.07†††
	**glutathione metabolism**					
	*Gsr*	Glutathione reductase	13.05±0.04	11.76±0.06	10.92±0.02**	12.94±0.05††
	*Gstm7*	Glutathione S-transferase, mu 7	110.40±0.07	107.17±0.04	86.55±0.10**	112.61±0.04††
	*Cflar*	CASP8 and FADD-like apoptosis regulator	2.13±0.08	2.09±0.08	1.45±0.19**	2.35±0.04†††
	*Plch1*	Phospholipase C, eta 1	0.56±0.27	0.48±0.19	1.17±0.10*	0.54±0.21††
**STR**	**TCA cycle**					
	*Idh3B*	Isocitrate dehydrogenase 3 (NAD+) beta	68.88±0.07	46.58±0.14†	46.54±0.17*	71.29±0.05†
	*Mdh1*	Malate dehydrogenase 1, NAD (soluble)	37.49±0.05	34.35±0.04	102.42±0.44**	38.26±0.04††
	*Ndufs4*	NADH dehydrogenase (ubiquinone) Fe-S protein 4	34.87±0.47	92.84±0.05†	90.32±0.03*	16.75±0.23†††
	*Plaa*	Phospholipase A2, activating protein	2.43±0.14	2.90±0.05	8.57±0.04***	3.10±0.30†††
	**Calcium signaling**					
	*Chp*	Calcium-binding protein p22	5.45±0.13	5.14±0.11	12.36±0.14***	6.80±0.21††
	*Ephb1*	Eph receptor B1	15.91±0.05	13.60±0.08	7.93±0.29***	12.83±0.12†
	*Letm1*	Leucine zipper-EF-hand-containing transmembraneprotein 1	63833.44±0.10	88257.60±0.11	105319.91±0.25*	64125.09±0.20†
	*Rap1b*	RAS-related protein 1b	24.66±0.09	22.30±0.13	13.56±0.30**	25.37±0.09††

The expression of genes in the pathways most significantly restored by nicotine in the prefrontal cortex (PFC), dorsal hippocampus (HIP), or dorsal striatum (STR) of HIV-1Tg rats. Data are expressed as the mean of fragments per kilobase of exon per million fragments mapped (FPKM) ± SEM in saline-treated F344 (F344_Sal), nicotine-treated F344 (F344_Nic), saline-treated HIV-1Tg (HIV_Sal), and nicotine-treated HIV-1Tg (HIV_Nic) rats.

* *p*<0.05,

** *p*<0.001,

*** *p*<0.001 indicate significant strain differences;

† *p*<0.05,

†† *p*<0.01,

††† *p*<0.001 indicate significant drug effects within each strain.

**Figure 3:**
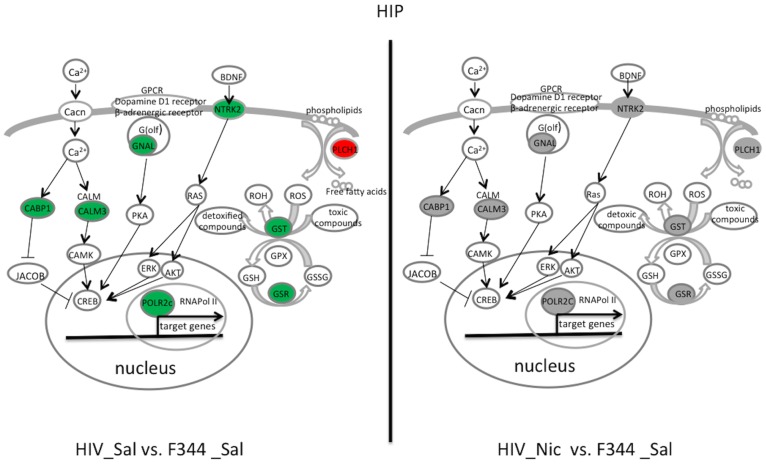
Illustration of top two pathways and related genes restored by nicotine in the dorsal hippocampus (HIP) of HIV-1Tg rats. Restored genes and pathways in saline-treated (HIV_Sal, left panel) and nicotine-treated (HIV_Nic, right panel) HIV-1Tg rats are shown. Compared with saline-treated F344 (F344_Sal) control rats, genes shown in red were increased, those shown in green were decreased, and those in gray had similar mRNA expression. AKT, v-akt murine thymoma viral oncogene homolog; CABP1, calcium-binding protein 1;Cacn, Ca^2+^ channel; CALM, calmodulin; CALM3, calmodulin 3; CAMK, calcium/calmodulin-dependent protein kinase; CREB, cAMP-responsive element-binding protein; BDNF, brain-derived neurotrophic factor; ERK, mitogen-activated protein kinase; G(olf), guanine nucleotide-binding protein complex, olfactory type; GNAL, guanine nucleotide-binding protein, alpha stimulating, olfactory type; GPX, glutathione peroxidase; GSH, glutathione; GSR, glutathione reductase; GST, glutathione-S-transferase; GSSG, glutathione disulfide; NTRK2, neurotrophic tyrosine kinase receptor, type 2; PKA, protein kinase A; PLCH1, phospholipase C, eta 1; POLR2C, polymerase (RNA) II polypeptide C; ROH, reduction product of reactive oxygen species; ROS, reactive oxygen species.

In the HIP (Figure 3, Table 2), the genes involved in CREB signaling, including calcium-binding protein 1 (*Cabp1*), calmodulin 3 (*Calm3*), guanine nucleotide-binding protein, alpha stimulating, olfactory type (*Gnal*), neurotrophic tyrosine kinase receptor, type 2 (*Ntrk2*), and polymerase (RNA) II (DNA-directed) polypeptide C (*Polr2c*) showed significantly less expression in the HIV_Sal group than in the F344_Sal group. The mRNA expression of all these genes were altered by nicotine in the HIV-1Tg rats, whereas only *Cabp1* was significantly decreased by nicotine in the F344 rats. These genes were all restored by nicotine to normal in the HIV-1 Tg rats, as there was no significant difference between HIV_Nic and F344 _Sal rats. Glutathione metabolism-related genes, including glutathione reductase (*Gsr*), glutathione S-transferase, mu 7 (*Gstm7*), and CASP8 and FADD-like apoptosis regulator (*Cflar*), were downregulated and phospholipase C, eta 1 (*Plch1*) was upregulated in the HIP of HIV_Sal compared with F344_Sal group. All of these genes were significantly changed by nicotine treatment in the HIV-1Tg but not F344 rats. After nicotine treatment, the expression of all these genes in the HIV-1Tg rats was similar to that in the F344_Sal group.

**Figure 4:**
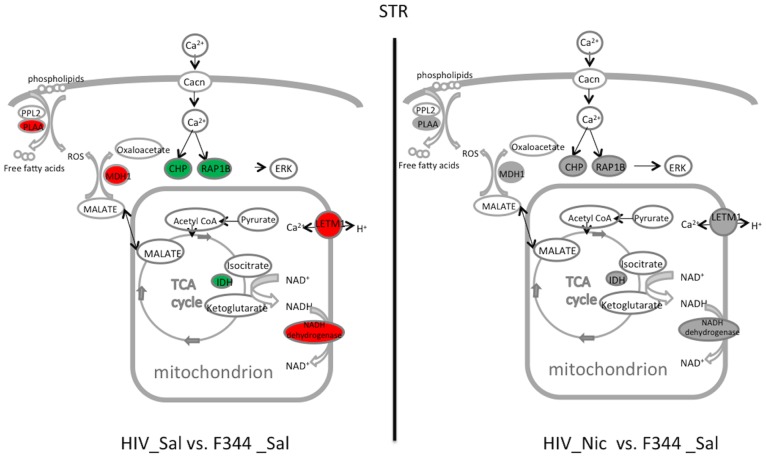
Illustration of top two pathways and related genes restored by nicotine in the dorsal striatum (STR) of HIV-1Tg rats. Restored genes and pathways in saline-treated (HIV_Sal, left panel) and nicotine-treated (HIV_Nic, right panel) HIV-1Tg rats are shown. Compared with saline-treated F344 (F344_Sal) control rats, genes shown in red were increased, those shown in green were decreased in expression, and those in gray showed similar mRNA expression. Cacn, Ca^2+^ channel; CHP, ERK, mitogen-activated protein kinase; IDH, isocitrate dehydrogenase; LETM1, leucine zipper-EF-hand-containing transmembrane protein 1; MDH1, malate dehydrogenase 1, NAD (soluble); PLAA, phospholipase A2, activating protein; PPL2, phospholipase 2; RAP1B, RAS related protein 1b; ROS, reactive oxygen species.

In the STR (Figure 4 and Table 2), the mRNA expression of TCA cycle-related genes, including malate dehydrogenase 1, NAD (Mdh1), NADH dehydrogenase (ubiquinone) Fe-S protein 4 (*Ndufs4*), and phospholipase A2 activating protein (*Plaa*) were upregulated, while isocitrate dehydrogenase 3 (NAD+) beta (*Idh3B*) was downregulated in the HIV_Sal compared with F344_Sal rats. The expression of *Idh3B* was increased by nicotine in the HIV-1Tg rats while it was decreased in the F344 rats. In contrast, *Ndufs4* was downregulated by nicotine treatment in the HIV-1Tg rat, whereas it was upregulated by nicotine in the F344 rats. Both *Mdh1* and *Plaa* were downregulated by nicotine in the HIV-1Tg rats but were not changed by nicotine in the F344 rats as compared to their saline treated controls. After nicotine treatment, the expression of all of these genes showed no differences between HIV-1 Tg and F344_Sal rats. In calcium signaling, the expression of calcium-binding protein p22 (*Chp*) and leucine zipper-EF-hand-containing transmembrane protein 1 (*Letm1*) were increased, whereas the expression of Eph receptor B1(*Ephb1*) and RAS-related protein 1b (*Rap1b*) was decreased in the HIV_Sal compared with the F344_Sal group. Nicotine decreased the expression of *Chp* and *Letm1*, and increased the expression of *Ephb1* and *Rap1b*, in the HIV-1Tg rats, whereas it had no effect on the expression of any of these genes in the F344 rats. After nicotine treatment, all of these genes in the HIV-1Tg rats were expressed to a degree similar to that in the F344_Sal group.

## Discussion

Studies of the effects of nicotine on HIV-1 pathogenesis are still few. Our exon-wide gene expression analyses showed that nicotine, at least partially, reversed abnormal gene expression in the CNS of HIV-1Tg rats. This finding is consistent with those of previous studies suggesting that HIV-1 proteins and nicotine have opposite effects by regulating their shared pathways in opposite directions [18], [21], [41].

By evaluating brain regions important in cognition and motor control, we found that nicotine restored the gene expression in the HIV-1 Tg rats in a brain region-dependent manner. Although the mechanisms underlying the brain region differences are still less clear, it may result from expression of different nicotinic receptors and distinct neurochemistry [50], [51].

### Prefrontal Cortex

Among the pathways restored by nicotine in the PFC of HIV-1Tg rats, Wnt/β-catenin signaling was the most significant. Such signaling, also known as canonical Wnt signaling, is activated by the binding of Wnt ligands to the frizzled membrane receptors and their co-receptors (LRP5/6), which prevents degradation of β-catenin. The stabilized β-catenin enters the nucleus and regulates gene transcription [52]. Our data demonstrated that the abnormal expression of a few genes of Wnt/β-catenin signaling in the HIV-1Tg rats, including the downregulation of *Wnt5a*, *Wnt7a*, and *Axin1* and the upregulation of *Gnao1*, was restored by nicotine to a level similar to that in saline-treated F344 rats. Moreover, we observed that the decreased expression of Fgf9, a fibroblast growth factor with neuroprotective function [53], in the HIV-1Tg rats was increased by nicotine treatment. Wnt/β-catenin signaling plays a major role in the early development of the nervous system, and activation of this pathway has a neuroprotective effect in adults [54], [55]. Recent studies have shown that Wnt/β-catenin signaling is also essential in myelin gene expression and myelinogenesis [56], which is important in neuronal survival [57]. Therefore, restoration of Wnt/β-catenin pathways and related genes suggests that nicotine can enhance neuronal survival in the PFC of HIV-1Tg rats. This effect is important since neurodegeneration and central demyelination have been documented in HIV-1 infected patients and animal models [58]–[60] and related to neurocognitive deficits [6]. Our data also showed that the expression of a few genes regulating the function of NMDA receptor was restored by nicotine in the HIV-1 Tg rats. Firstly, the expression of Wnt5a, which positively regulates synaptic plasticity and NMDA-mediated glutamatergic transmission via the non-canonical pathways [61]–[63], was increased to normal by nicotine in the HIV-1 Tg rats Secondly, ephrin B and ephrin receptor signaling, which regulate synaptic formation and plasticity by interacting with NMDA receptors [64]–[67], was significantly restored by nicotine. Thirdly, the decreased expression of *Mapk8ip2*, a scaffold protein facilitating the activity of the NMDA receptor [68], in the HIV-1 Tg rats was increased by nicotine treatment. Our previous study has shown obvious deficits in learning and memory in the HIV-1Tg rats [16], [43]. Given that neuroplasticity and NMDA-mediated glutamatergic transmission in the PFC is important in learning and memory [69], our data indicate that nicotine improves PFC-mediated learning and memory in HIV-1Tg rats by restoring the expression of genes modulating NMDA receptor activity.

### Hippocampus

Our pathway analysis showed that CREB signaling was most significantly restored by nicotine in the HIP of HIV-1Tg rats. The CREB proteins are a class of transcription factors that bind to selected cAMP-response element (CRE) segments of DNA and catalyze the transcription of these genetic sequences. HIV-1Tg rats showed decreased expression of Calm3 and Cabp1which are calcium sensor proteins activating CREB signaling in a Ca^2+^-dependent manner [63], [70], [71]. The expression of these genes was normal following nicotine treatment. Furthermore, nicotine increased the expression of Gnal, a subunit of G protein [G(olf)], in the HIV-1 Tg rats. G(olf) is well known to regulate signal transduction of G(olf)-coupled receptors such as the dopamine D1 and -adrenergic receptors and thus induces gene expression through CREB signaling [72], [73]. We also observed the expression of Ntrk2, the receptor for BDNF/NT-3, was increased in nicotine-treated HIV-1Tg rats. Ntrk2 regulates CREB activity by several mechanisms, including via the ERK pathway [74]. Together, our data indicate a downregulation of CREB signaling in HIV-1Tg rats, which is consistent with a previous study showing decreased CREB phosphorylation in the brain of this transgenic model [18]. Our data further suggest that nicotine restores CREB-dependent gene regulation by improving signaling from Ca^2+^, G-protein-coupled receptors and growth factors. CREB signaling is critical in long-term synaptic plasticity and neuronal survival and therefore has been implicated as an important regulator of long-term memory [75], [76]. Nicotine therefore may improve HIP-dependent learning and memory by restoring CREB signaling in the HIP of HIV-1Tg rats.

HIV-1-infected patients have a higher incidence of oxidative stress, and animal studies show that HIV-1 viral proteins induce oxidative stress in the cardiovascular system [74], [75]. Consistent with these studies, our data indicate increased oxidative stress in the brains of HIV-1 Tg rats. This is reflected by abnormal glutathione metabolism, which is important in coping with oxidative stress, in the HIV-1Tg rats. Furthermore, we found that glutathione metabolism was significantly restored by nicotine in the HIP, with increased expression of GSR and GSTM2 in the nicotine-treated compared with saline-treated HIV-1Tg rats. Glutathione is one of the most abundant antioxidants in the brain and protects cells from oxidative stress-induced damage. Both GSR and GSTM7 are important enzymes, catalyzing glutathione recycling and its detoxification, respectively [77]. A decreased amount of glutathione and abnormal activities of these enzymes have been associated with aging and many neurodegenerative diseases, including AD and PD [78]–[80]. Therefore, restoration of glutathione metabolism by nicotine may protect neurons from oxidative stress-induced degeneration.

Oxidative stress also can be induced by overactivation of phospholipases, which regulate the turnover of free fatty acids in membrane phospholipids [81]. We observed overexpression of a phospholipase C (Plch1) in the HIV-1Tg rats, which was corrected by nicotine treatment. In addition, we noted that the expression of Cflar, a member of the death effector domain family [82], was restored by nicotine in the HIV-1Tg rats. These data further suggest that nicotine exerts a neuroprotective effect in the HIP of HIV-1Tg rats by decreasing oxidative stress.

### Striatum

In the STR of HIV-1Tg rats, the tricarboxylic acid (TCA) cycle was the most significant pathway restored by nicotine. The TCA cycle, also known as the citric acid or Krebs cycle, is the major pathway for energy generation. In the mitochondria of eukaryotic cells, the TCA cycle and its closely linked pathway, the oxidative phosphorylation pathway, oxidize nutrients to produce usable energy in the form of ATP. Our data suggest that nicotine restores the function of the TCA and its related pathways. Firstly, we discovered downregulation of the expression of isocitrate dehydrogenase (*Idh3B*), which catalyzes the rate-limiting step of the TCA cycle, in the HIV-1Tg rats. This alteration was reversed by nicotine treatment. Secondly, a NADH dehydrogenase (*Ndufs4*), which catalyzes the first step of oxidative phosphorylation, was overexpressed in the HIV-1 Tg rats but decreased to normal after nicotine treatment. This suggests overactivation of oxidative phosphorylation, which may compensate an impaired TCA cycle in the STR of HIV-1Tg rats. However, overactivaton of oxidative phophorylation increases the chance of oxidative stress. The increased oxidative stress was further supported by the increased expression of malate dehydrogenase (Mdh1), an enzyme catalyzing the oxidation of malate, and overexpression of phospholipase activating protein (Plaa), which activates phospholipase 2 to modulate the turnover of free fatty acids in membrane phospholipids [81]. The expression of all of these genes was corrected by nicotine. Therefore, our data suggest that nicotine restores the function of the TCA cycle, and reduces oxidative stress in the STR of HIV-1Tg rats, thereby enhancing neuronal survival in that brain region.

Abnormal calcium signaling has been suggested as one of the mechanisms underlying HIV-1-induced neurodegeneration [83]–[85]. Our data showed that calcium signaling was significantly restored by nicotine in the STR of HIV-1Tg rats. Ephb1, which modulates NMDA receptor-dependent calcium influx [67], was downregulated in the STR of HIV-1Tg rats. Whereas Letm1, a mitochondrial Ca^2+^/H^+^ antiporter [86], was overexpressed in the HIV-1Tg rats. Over-expression of Letm1 dramatically accelerate Ca^2+^ uptake into mitochondria [86]. Therefore, alterations in the expression of these genes indicate a decrease in cytoplasmic Ca^2+^ transduction in the STR of HIV-1Tg rats. Moreover, the expression of a few Ca^2+^-activated proteins such as Rap1b and Chp was abnormal in the HIV-1Tg rats. The mRNA expression of all these genes was reversed by nicotine in HIV-1 Tg rats, indicating restoration of Ca^2+^ signal transduction in the STR of HIV-1Tg rats. Because the STR is important in motor control, learning, and memory, nicotine may have beneficial effects on those functions by restoring important signal transduction in the STR of HIV-1Tg rats.

In addition to the brain region differences, we also noticed strain differences in response to nicotine with most of the genes altered by nicotine in HIV-1Tg rats were not significantly changed in F344 rats and some genes even showed opposite changes by nicotine as compared to that in HIV-1Tg rats. Although the underlying molecular mechanisms are less clear, such strain differences were unlikely due to different mRNA expression of nicotinic acetylcholine receptors as shown in our previous study [60]. This suggests that nicotine at least at the current dose has distinct neurobehavioral effects on normal and HIV-1Tg rats.

### Conclusions

**Figure 5:**
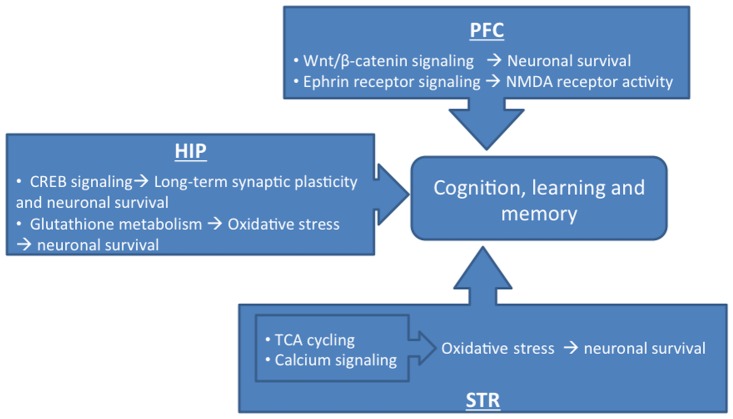
Summary of the pathways most significantly restored by nicotine in the prefrontal cortex (PFC), dorsal hippocampus (HIP), and dorsal striatum (STR) of HIV-1Tg rats and the potential effects of the restorations on those rats.

Using exon-wide gene expression analyses, we demonstrated that nicotine partially corrected the abnormal gene expression and altered signaling pathways in the CNS of HIV-1Tg rats. Although this study examined only one dose of nicotine, our data strongly indicate that nicotine has beneficial effects on neurologic problems associated with HIV-1 infection, especially in patients in which viral replication is controlled, but viral proteins persist. Nicotine appeared to have neuroprotective effects by restoring distinct signaling pathways in different brain regions, thus improving cognition, learning, and memory (Figure 5). Our findings also indicate that, in addition to nicotine, other cholinergic modulators may provide potential treatments for neurologic problems associated with HIV-1 infection.
